# Strategic management in local hospital markets: service duplication or service differentiation

**DOI:** 10.1186/s12913-020-05728-y

**Published:** 2020-09-17

**Authors:** Hanh Q. Trinh

**Affiliations:** Health Care Administration, University of Wisconsin-Milwaukee, Northwest Quadrant B 6428, 2025 E Newport Avenue, Milwaukee, WI 53211-2906 USA

**Keywords:** Hospital services, Hospital strategy, High-tech services, Service duplication, Service differentiation

## Abstract

**Background:**

The purpose of this study is to assess the influences of market structure on hospitals’ strategic decision to duplicate or differentiate services and to assess the relationship of duplication and differentiation to hospital performance. This study is different from previous research because it examines how a hospital decides which services to be duplicated or differentiated in a dyadic relationship embedded in a complex competitive network.

**Methods:**

We use Linear Structural Equations (LISREL) to simultaneously estimate the relationships among market structure, duplicated and differentiated services, and performance. All non-federal, general acute hospitals in urban counties in the United States with more than one hospital are included in the sample (*n* = 1726). Forty-two high-tech services are selected for the study. Data are compiled from the American Hospital Association Annual Survey of Hospitals, Area Resource File, and CMS cost report files. State data from HealthLeaders-InterStudy for 2015 are also used.

**Results:**

The findings provide support that hospitals duplicate and differentiate services relative to rivals in a local market. Size asymmetry between hospitals is related to both service duplication (negatively) and service differentiation (positively). With greater size asymmetry, a hospital utilizes its valuable resources for its own advantage to thwart competition from rivals by differentiating more high-tech services and reducing service duplication. Geographic distance is positively related to service duplication, with duplication increasing as distance between hospitals increases. Market competition is associated with lower service duplication. Both service differentiation and service duplication are associated with lower market share, higher costs, and lower profits.

**Conclusions:**

The findings underscore the role of market structure as a check and balance on the provision of high-tech services. Hospital management should consider cutting back some services that are oversupplied and/or unprofitable and analyze the supply and demand in the market to avoid overdoing both service duplication and service differentiation.

## Background

The competitive market facing hospitals in urban areas is very different from other markets. Each hospital is large enough to have considerable influence on the market. Because there are only a few hospitals serving the same clientele in the market, they are interdependent and closely watch one another’s moves [1]. As they compete for the same local clientele, sustaining market share is their primary concern. It is a zero-sum game where, if one gains market share, others lose. To sustain market share, hospitals usually engage in two fundamental strategic activities around their service offerings – service duplication and service differentiation.

The first strategy involves duplicating services that are already offered by rivals in an attempt to attract customers away from them [2–5]. This kind of competition among local hospitals can lead to a “medical arms race,” and eventually to a price war, where hospitals end up as either winners or losers. The second strategy is service differentiation, which avoids competition based on prices [6–9]. Hospitals may provide services that others do not offer, thus making themselves different from their rivals. They may be able to demonstrate the competence and high proficiency needed to control or to bring down the cost of these services [10]. While not a contributor to a medical arms race, the differentiation strategy can encourage hospitals to offer services that may not be justified economically.

The purpose of this paper is to study what influences hospitals in their pursuit of either service duplication or service differentiation, and the impact of their strategic activities on hospital performance. We focus on high-tech services because these represent a special segment of a local market that has the potential for creating differentiation as well as duplication. This study is different from previous research because it examines how a hospital decides which services to be duplicated or differentiated in a dyadic relationship embedded in a complex competitive network, and because it examines the relationship of both strategies to performance.

### Framework and hypotheses

The institutional theory of organizations argues that firms often will better position themselves in a market by duplicating services (a form of imitation) that are offered by rivals [11, 12]. A firm uncertain of customer needs tends to imitate the practices of successful firms, assuming its rivals are accepted in the competitive market. The advantage of offering similar services is to gain legitimacy while avoiding the risk of making the first move [13–15]. If a firm is not sure what the best course of action is, imitating others is a way to maintain its relative position or to neutralize the action of rivals [16]. On the other hand, there is a risk that competition can be very intense, eroding prices and profits [17]. It is common to see hospitals in the general acute service market providing similar services such as general medicine, obstetrics, cardiology, pediatric, etc.

In addition, cost-based reimbursement historically has motivated hospitals to compete without considering the costs of care, offering a full range of services and latest technologies to attract physicians and patients [18–20]. Under cost-based reimbursement, hospitals have incentives to offer services with little regard for economic efficiency even when the services are duplicated across hospitals [18]. Previous studies have focused on the effects of duplicated services on hospital performance, and the environmental and organizational characteristics that influence service duplication [2–5]. Other studies have focused on the service offerings of hospitals clustered in the same multihospital system. They found that consolidation of services lags behind consolidation of administrative services, which suggests that medical staff are reluctant to change practice patterns and hospitals prefer to continue to preserve revenues [21–35]. These forces uphold the offering of duplicative services across cluster members.

The strategic management theory suggests that firms can also compete by providing services that rivals do not have, a form of differentiation. By being different, a firm can benefit as it faces less competition, earns higher revenues, and has a good chance to run a local monopoly [36–38]. There are many ways hospitals can differentiate their services. Some focus on high-tech services and use of the latest technologies such as robot surgery, or organ transplant. Some focus on quality of medical staff. Others pursue rare services such as burn care, proton therapy, and magnetoencephalography (MEG). Both duplication and differentiation are associated with benefits and costs that can have a profound impact on hospital performance in market share, cost of services, and profits.

Service differentiation has received some attention as a means of classifying hospital system types [39, 40], and differentiation of services has been associated with several factors, most notably higher market competition [9, 19, 41]. However, its relationship with performance remains relatively unexplored.

One of the key arguments we propose in this study is that geographical proximity induces rivalry, which, in turn, leads to either duplication or differentiation of services. Economic theory suggests that firms located far distant from each other will face less competition. When they are closer to each other geographically, competition becomes fierce [42]. In a hospital market, when hospitals locate far apart, competition for similar high-tech services is less intense because distance serve as an effective barrier, sorting out real from potential rivals [1]. When there are no nearby rivals (e.g., within 15 miles), hospitals face less direct competitive threat, as nearby customers have convenient access to their services. If firms locate near each other, however, they may avoid providing similar services from rivals because similarity forces them into a price war that squeezes profit. They turn to differentiation of services to reduce competition. The above discussion suggests the following hypotheses.

#### Hypotheses 1–2

*Hospitals duplicate more services of rivals if their location is geographically farther apart. Hospitals differentiate more services from rivals if their location is geographically proximate.*

Another key factor influencing the choice of duplication or differentiation is asymmetry among firms. Not all firms possess the same level of resources. Some firms have unique and valuable resources, which allow them to sustain competitive advantage and enhance opportunism to expand market share, deliver services that are most profitable, and leave services that are imitable to weaker firms. Power asymmetry exists when a firm uses its power, through its valuable resources, to dominate or influence the actions of other firms in a dyadic relationship for its own advantages [43]. The asymmetrical relationship puts a weaker firm at greater degree of vulnerability to opportunism. The powerful firm may use its advantage to maximize its capabilities that distinguish it from its rivals [38]. It also uses its advantage to gain more leverage at the expense of the weaker firm, and coerces the weaker firm to perform tasks on its behalf [44].

In a local hospital market, a powerful hospital with special capabilities usually provides customers with high-tech services that are not only inimitable but also more profitable than other imitable services. Inimitable high-tech status is not easy to deliver as it is a combination of possession of knowledge, use of the knowledge, and proficiency of its use [45]. A hospital with these capabilities would leverage its power advantage to oppose or circumvent competition, whatever it decides to do [46]. Since the dominant hospital substantially holds more capabilities than the others, it establishes the power asymmetry which eventually is accepted by other hospitals in the market [1]. A dominant hospital tends to exercise its potential capabilities to differentiate high-tech services, while a weaker hospital duplicates services that are imitable. We therefore hypothesize the following.

#### Hypotheses 3–4

*Hospitals with more power asymmetry differentiate more services from rivals. Hospitals with less power asymmetry duplicate more services of rivals.*

Strategic management theory suggests that market competition is a key component that induces firm to protect market share from rivals. Because firms in the same industry located in a local market are mutually dependent, the strategy of one firm will affect that of rivals. As competition is mounting, a firm must position itself vis-à-vis its rivals, and it looks for ways to differentiate its services from the rest. With differentiation, firms circumvent duplication of services. By offering different services, a firm reduces competition and avoids competition based on price. In a hospital market, market competition is likely to influence hospitals to protect their market share with service differentiation [9, 47].

#### Hypotheses 5–6

*Hospitals in more competitive markets duplicate fewer services of rivals and differentiate more services from rivals.*

In addition to geographical proximity, power asymmetry, and market competition, other market forces also influence firms’ strategic behavior. In the health care industry, other market pressures notably come from population density, community munificence, specialist physicians, and managed care. In a community with high population density, there are naturally more patients with medical problems that require more high-tech services. Located in such a high- density community, hospitals are likely to provide as many services as possible. They tend to duplicate as well as differentiate services from rivals.

#### Hypotheses 7–8

*Hospitals in communities with higher population density duplicate and differentiate more services from rivals.*

Community munificence plays a role in firm strategies as it is the resources that support firm growth. Firms located in a community with high munificence have more advantages to survive [48]. These firms are able to pursue exploration strategies for new services to make them unique. Wealthy patients in a highly munificent community have ample financial resources to pay for expensive high-tech services, and hospitals are more likely to meet their needs.

#### Hypotheses 9–10

*Hospitals in wealthier communities duplicate and differentiate more services from rivals.*

To compete with rivals, firms will try to attract the most talent to work for them. Likewise, hospitals tend to offer high-tech services as an inducement to attract the most talented physicians, particularly specialist physicians [3, 49].

#### Hypotheses 11–12

*Hospitals in communities with higher density of specialist physicians duplicate and differentiate more services from rivals.*

To attract buyers, firms are more likely to present themselves as purveyors of high-quality services. In the health care industry, insurance companies demand high-tech services on behalf of their members. Hospitals in a community with greater managed care penetration are more likely to provide high-tech services to attract customer business as a way of maintaining or increasing market share [3, 50].

#### Hypotheses 13–14

*Hospitals in communities with more managed care presence duplicate and differentiate more services from rivals.*

As other characteristics from the organization might influence firms’ strategic behaviors, we include them in our study as control variables. They are multihospital membership, non-profit ownership, bed size, and case mix index. These variables could influence the levels of service duplication and service differentiation.

Economic theory suggests that firms with valuable assets that are rare, inimitable and difficult to copy will be able to pursue strategic differentiation. These firms will face less competition and thus will have a good market share with a better profit. In a local market, firms closely watch rivals’ move, and such a strategic position will soon be copied by others. They are attracted to this profitable opportunity and are eager to join in the good fortunes of the successful firms [16]. But imitation is associated with costs as well as with benefits. The benefits firms can enjoy are the legitimacy and access to the market. The cost is the erosion of profitability. As some firms imitate the successful firms, imitation of products can lead to competition with lower prices [16]. As more firms offer the same or similar services, they sooner or later engage in direct competition through which a price war emerges. Lower prices will unavoidably squeeze profits to the level that no firms will be able to make extra profits. However, firms can avoid this unexpected situation if they find ways to collude. Empirical research indicates that firms offering similar services often recognize their interdependence and collude [51–53].

In a local hospital market, offering different services has both benefits and costs. With a wide range of high-tech services that are inimitable like robot surgery and organ transplantation, hospitals are able to increase market share by attracting more customers for more revenues. With the increase in usage of these services, cost per unit will fall [10, 54]. However, they might not be able to maximize economies of scales if their high-tech services are too expensive for consumers to afford. On the other hand, duplicating services might not gain adequate volumes of services for lowering cost per unit. It also might inevitably force hospitals to engage in a price war that leads to lower profits.

#### Hypotheses 15–16

*Hospitals differentiating more services will have higher market share, higher profits and lower costs. Hospitals duplicating more services will have lower market share, lower profits, and higher costs.*

## Methods

### Data set and sample

In this study, a non-federal general acute hospital in an urban county in the United States is the unit of analysis. “Urban” is defined as an area located inside the United States Census Bureau’s “Core Based Statistical Areas” (CBSAs). The data on high-tech service duplication and differentiation were drawn from the 2015 American Hospital Association’s (AHA) Annual Survey of Hospitals file. Data on organizational characteristics and environmental factors were obtained from the 2015 AHA and the 2015 Area Resource File. Data on managed care were retrieved from the 2015 HealthLeaders-InterStudy source.

The data set consists of 1726 general acute hospitals, compared to a total of 2831general acute hospitals in 2015 in urban counties. There are two reasons for this difference. First, 830 hospitals are sole hospitals that do not exercise a strategy of service similarity or differentiation because there are no other hospitals in their county for competition. Second, 246 hospitals are reported as acute in the AHA file but as non-acute in CMS data files. Third, 29 hospitals do not report assets. Therefore, they were excluded from our sample.

Different from previous studies that focus on hospitals’ broader competitive responses in a market, this study calls for attention to the dyadic competition between two rivals where the influences of geographical distance together with power asymmetry, market competition, multi-hospital membership are more evident. As two rivals are embedded in a web of competitive relationships with other rivals where these two rivals respond not only to each other’s moves, but also to other rivals through a chain of interactions in the market, they make strategic decisions with uncertain and incomplete knowledge. The complicated, intertwined competition embedded in the web create a big challenge for hospitals to develop effective strategies.

A way to untangle strategic decisions to duplicate or differentiate from rivals is to examine each decision in a dyad. For example, consider a market with 15 services, in which hospital A provides services 1, 2, 3, 4, 5, and 6; hospital B services 5, 6, 7, and 8; hospital C services 9, 10, 11, 12, 13, 14, and 15. Hospital A may want to provide service 9 in order to differentiate from hospital B, but hospital C already provides service 9. In other words, hospital A is not able to differentiate service 9 in relation to hospital C. If hospital A chooses to differentiate service 9 in relation to hospital B, it will duplicate service 9 with hospital C. As all hospitals one by one continue adding more services for differentiation, they might unintentionally turn the process of differentiation into a process of duplication. The advantage of our methodological approach is that it reflects the reality in which firms respond to each of rivals’ moves [19]. It allows us to have a better understanding why firms decide to duplicate or differentiate services with some rivals, but not with others.

This study defines duplication as number of the same services offered by the focal hospital in a dyad, and differentiation as the number of unique services offered by the focal hospital in a dyad. The algorithm first creates all possible pairs of hospitals in each county, then counts the number of services each hospital offers that each of its potential rivals do or do not. The number of possible pairs of hospitals is calculated using a concept of permutation [55] as follows:

$$ {{}_{\mathrm{n}}\mathrm{P}}_{\mathrm{r}}=\frac{\mathrm{n}!}{\left(\mathrm{n}\hbox{-} \mathrm{r}\right)!} $$

P: number of possible pairs of hospitals.

n: number of hospitals located in the same county.

r: number of hospitals in each pair.

With this permutation approach, the number of possible pairs of hospitals increases substantially with an increase in the number of pairs of hospitals within the same county. For example, there are two possible pairs (AB and BA) with two hospitals A and B in the same county, six possible pairs (AB, BA, BC, CB, AC, and CA) with three hospitals A, B, and C, etc. By adding up all these possible pairs, the sample size for the study increases from 1726 to 11,264. Because the permutation of a set of hospitals is an ordered sequence, it means that AB and BA are two different possible pairs. Within each possible pair, the first hospital is treated as a focal one, and the second is its potential rival. Services provided by these two hospitals are compared to identify which services the second hospital provides or does not provide. These services are the ones that the focal hospital duplicates or differentiates from the potential rival. For example, suppose hospital A provides services 1–6, and hospital B offers services 5–8. Hospitals A and B provide the same services 5 and 6, but they differentiate from each other on other services. Hospital A differentiates with services 1, 2, 3, and 4, and hospital B with services 7 and 8.

We transformed the data of 1726 hospitals in a vector form into a new dataset with a matrix form using the Structured Query Language (SQL) procedure in SAS to produce 12,364 possible pairs of hospitals in which a focal hospital is paired with each of its rivals in the same county. With this new data set, we calculated matrix variables such as geographical distance, power asymmetry, similar and different services before saving them along with other variables in another dataset for further analysis.

## Measurement

### Exogenous variables

#### Market structure variables

##### Geographical distance

Geographical distance is the straight-line distance between the focal hospital and its potential rival. Geographic Information System (arcGIS) is used to calculate the distances between pairs of hospitals.

##### Size asymmetry

Size is used as a measure of power. Size asymmetry is measured as a ratio of bed size of a focal hospital to the bed size of its potential rival.

##### Local market competition

Local market competition is measured at the county level with 1 - Herfindahl-Hirschman Index (HHI). HHI is the sum of the squared proportions of each hospital’s bed size to total bed size within the same county.

##### Population density

Population density is measured as a population of the county in thousands divided by the area in square miles.

##### Community munificence

Munificence is measured as income in thousands divided by the population of the county.

##### Specialist physicians

Specialist physician density is measured by total specialist physicians per 1000 population in the county.

##### Managed care

Managed care is measured as the ratio of the number of people covered by managed care in the state to the state population (due to the unavailability of this measure at the county level).

#### Control variables

##### Multihospital system membership

Membership is measured with 0 representing no membership, and 1 representing membership in a multihospital system.

##### Non-profit ownership

Ownership status is measured with 1 representing for-profit, and 0 representing non-profit.

##### Bed size

Bed size is measured as the number of staffed beds.

##### Case mix index

Case mix index is the degree of severity of illness, prognosis, treatment difficult, and resource intensity. The index is from the CMS file 2015.

#### Endogenous variables

Three endogenous variables are used to measure hospital performance, as follows.

##### Market share per bed

Market share per bed is the hospital’s patient days divided by the county’s total patient days. This ratio is divided by the hospital’s number of beds.

##### Cost per discharge

Cost per discharge is used to capture hospital costs associated with delivering health care. It is the total cost divided by the number of cases. Because hospitals have different case mix severity, the cost per discharge is adjusted with case mix index so that comparison across hospitals can be made.

##### Return on assets

Commonly used in studies of financial performance, return on assets is the profit/loss divided by total assets. Profit/loss is the difference between revenues and expenses. Return on assets measures the ability to use company assets to generate profits.

The final endogenous variables are the number of duplicated and differentiated services.

##### Duplicated services and differentiated services

As noted earlier, duplication and differentiation are measured by pairing a focal hospital with each of its potential rivals one at a time. With each pairing, duplication is the number of high-tech services that a focal hospital and its rival both have, and differentiation is the number of high-tech services that a focal hospital has but its potential competitor does not. This measurement makes it possible to understand which market factors influence the way hospitals decide to provide the similar or different services.

Table 1 includes 42 services (see Table 1) reported in the AHA data file that require high-technology, such as organ transplant, magnetic resonance imaging, cardiac surgery, robotic surgery, to capture a wide range of services [56]. They are the type of services utilized by hospitals in their efforts to differentiate themselves from competitors [8, 47, 54].

**Table 1:** Hospital High-Tech Services

1. Computed-tomography (CT) scanner	
2. Magnetic resonance imaging (MRI)	
3. Diagnostic radioisotope facility	
4. Optical Colonoscopy	
5. Full-field digital mammography	
6. Multi-slice spiral computed tomography < 64 slice	
7. Multi-slice spiral computed tomography 64 + slice	
8. Endoscopic retrograde	
9. Adult diagnostic/invasive catheterization	
10. Single photon emission computerized tomography	
11. Adult interventional cardiac catheterization	
12. Endoscopic ultrasound	
13. Adult cardiac electrophysiology	
14. Extracorporeal shock-wave lithotripter (ESWL)	
15. Robotic surgery	
16. Adult cardiac surgery	
17. Intensity-Modulated Radiation Therapy (IMRT)	
18. Ablation of Barretts esophagus	
19. Esophageal impedance study	
20. Image-guided radiation therapy	
21. Positron emission tomography/CT (PET/CT)	
22. Shaped beam Radiation System	
23. Stereotactic radiosurgery	
24. Positron emission tomography (PET)	
25. Computer assisted orthopedic surgery	
26. Virtual colonoscopy	
27. Genetic testing/counseling	
28. Tissue transplant	
29. Electron Beam Computed Tomography (EBCT)	
30. Other transplant	
31. Kidney transplant	
32. Intraoperative MRI (IMRT)	
33. Pediatric card electrophysiology	
34. Bone Marrow transplant services	
35. Pediatric diagnostic/invasive catheterization	
36. Pediatric interventional cardiac catheterization	
37. Pediatric cardiac surgery	
38. Magnetoencephalography (MEG)	
39. Liver transplant	
40. Heart transplant	
41. Proton therapy	
42. Lung transplant	

Measures and sources are summarized in Table 2.

**Table 2:** Measures and Sources

VARIABLE	MEASURE	SOURCE
Hospital performance		
Market share per bed	Log of percentage of hospital inpatient days divided by total market inpatient days adjusted for hospital bed size	AHA Annual Survey
Cost per discharge	Total hospital inpatient costs divided by total hospital discharges adjusted for case mix index	Center for Medicare and Medicaid Services
Return on assets	Profit divided by total assets	Center for Medicare and Medicaid Services
Duplicated services	Number of high-tech services that both a focal hospital and its competitor provide	AHA Annual Survey
Differentiated services	Number of high-tech services that a focal hospital provides but its competitor does not	AHA Annual Survey
Market structure		
Geographic distance	Log of distance in miles between local hospital and each of its competitors in a local market	AHA Annual Survey, arcGIS
Size asymmetry	Log of ratio of local hospital bed size over each of its competitors in a local market	AHA Annual Survey
Market competition	1 – HHI within county	AHA Annual Survey
Population density	Log of population of county in thousands divided by areas in miles	Area Resource File
Community munificence	Log of per capita income in thousand within county	Area Resource File
Specialists per 1000 population	Log of number of specialist physicians per 1000 population	Area Resource File
Managed care penetration	Percentage managed care members over population in state	Bates White Economic Consulting
Control variables		
Multihospital membership	0: No, 1: Yes	AHA Annual Survey
Ownership for non-profit	0: No, 1: Yes	AHA Annual Survey
Bed size	Log of staffed beds	AHA Annual Survey
Case mix index	Medicare case mix index	Center for Medicare and Medicaid Services

### Analytic method

The study utilizes path analyses to evaluate the simultaneous inter-relationships among sets of exogenous and endogenous variables. The linear structural relations (LISREL) approach to path analysis is used in this study due to its ability to simultaneously examine relationships among endogenous variable. Variables with positively skewed distribution were logged. Descriptive statistics and correlation coefficients for all variables are reported in Table 3.

**Table 3:** Descriptive statistics and correlation matrix of study variables (*n* = 11,264)

	Mean	S.D.	1	2	3	4	5	6	7	8	9	10	11	12	13	14	15	16
1. Market share per bed in % (log)	−3.916	1.117	1.000															
2. Cost per discharge In thousands	9.028	.291	−.144	1.000														
3. Return on assets	.052	.163	.125	−.108	1.000													
4. Duplicated services	12.249	6.213	−.003	.037	−.036	1.000												
5. Differentiated services	6.239	6.465	−.063	.227	−.029	−.128	1.000											
6. Geographic distance in miles (log)	2.339	.954	−.271	.015	−.039	−.194	−.021	1.000										
7. Size asymmetry (log)	−.001	1.098	−.003	.053	−.032	−.010	.682	.001	1.000									
8. Market competition	.871	.135	−.849	.189	−.110	−.018	.038	.328	−.001	1.000								
9. Population density (log)	.571	1.106	−.589	.157	−.064	.179	.050	−.042	.004	.524	1.000							
10. Community munificence (log)	3.949	.207	−.199	.204	−.064	.139	.026	−.010	.004	.236	.4703	1.000						
11. Specialists per 1000 population (log)	.953	.430	−.197	.011	−.025	.277	.062	.230	.001	.121	.517	.558	1.000					
12. Managed care	.377	.163	−.410	.314	−.145	−.242	.039	.258	−.008	.403	.019	.011	−.238	1.000				
13. Multihospital membership	.806	.396	−.021	−.063	−.007	.066	.025	−.011	.048	.014	.001	−.016	.016	−.041	1.000			
14. Non-profit ownership	.776	.417	.080	.212	−.230	.237	.201	−.077	.184	−.063	.072	0.080	.167	−.044	−.025	1.000		
15. Bed size (log)	5.443	.809	−.189	.058	−.075	.512	.493	−.143	.678	.119	.216	.067	.220	−.023	.083	−.283	1.000	
16. Case mix index	1.651	.263	−.123	.072	.086	.275	.418	−.136	.289	.090	.044	.062	.124	.057	.001	−.032	.398	1.000

LISREL output provides a variety of goodness-of-fit measures for the entire model. Among these are (1) the chi -square goodness-of-fit measure and its related degrees of freedom and probability level, (2) the goodness-of-fit (GFI), (3) the adjusted goodness-of-fit index (AGFI), and (4) the root mean square residual (RMSR). Results of the LISREL analysis indicate that the model fits the data well. The probability of the fit is 100%, and the chi-square with 67 degrees of freedom is 33.45. The goodness-of-fit (GFI) is 1.00 and the root mean square residual (RMSR) is 0.0051.

## Results

Results in Table 4 indicate that 11 out of 14 effects of market structure are significant in the hypothesized directions with either service duplication or differentiation, with 6 effects associated with service duplication, and 5 effects associated with service differentiation. As hypothesized, service duplication is positively associated with geographic distance and community munificence, and is negatively associated with size asymmetry, and market competition. Duplication is negatively associated with specialist physicians and managed care in the opposite direction of the hypotheses. Service differentiation is positively associated with size asymmetry, population density, specialist physicians, and managed care, but is negatively associated with munificence. Only the munificence result is contrary to the hypothesized direction. All control variables are significant.

**Table 4:** Parameter estimates (gammas) for effects of market structure and control variables on duplicated and differentiated services (*n* = 11,264)

	Duplicated services		Differentiated services	
	Hypothesized	Actual	Hypothesized	Actual
Market structure				
Geographic distance	+	0.025 ^c^	–	0.010
Size asymmetry	–	−0.666 ^c^	+	0.650 ^c^
Market competition	–	−0.045 ^c^	+	0.010
Population density	+	−0.015	+	0.051 ^c^
Community munificence	+	0.116 ^c^	+	−0.042 ^c^
Specialists	+	−0.072 ^c^	+	0.019 ^a^
Managed care	+	−0.230 ^c^	+	0.034 ^c^
Control variables				
Multihospital membership		0.043 ^c^		0.018 ^c^
Non-profit ownership		0.174 ^c^		0.180 ^b^
Bed size		0.852 ^c^		0.137 ^c^
Case mix		0.143 ^c^		0.273 ^c^

^a^ Significant at the .05 level

^b^ Significant at the .01 level

^c^ Significant at the .001 level

Table 5 displays all 6 significant beta coefficient estimates relating duplication and differentiation to performance. Duplication is negatively related to market share and return on assets and positively related to cost per discharge, as hypothesized. The same findings hold for differentiation but are opposite to the directions hypothesized.

**Table 5:** Parameter estimates (betas) for effects of duplicated and differentiated services on hospital performance (*n* = 11,264)

	Market share		Return on assets		Cost per discharge	
	Hypothesized	Actual	Hypothesized	Actual	Hypothesized	Actual
Duplicated services	–	− 0.286 ^c^	–	− 0.041 ^c^	+	0.067 ^c^
Differentiated services	+	−0.10 ^c^	+	−0.034 ^c^	–	0.236 ^c^

^c^ Significant at the .001 level

## Discussion

This study is different from previous research because it examines how a hospital decides which services to be duplicated or differentiated in a dyadic relationship embedded in a complex competitive network. Rather than using a gross measure as the number of duplicated or differentiated services that a hospital provides, we use more fine-tuned measures by counting the number of services that are duplicated or differentiated between a focal hospital and each of its rivals one at a time. The findings provide partial support for 8 of the 14 hypotheses that offer explanations for duplicating and differentiating arrangements among rivals in a local market. Size asymmetry has stronger effects than geographic distance and market competition as it influences both duplication and differentiation. Geographical distance appears to play a role in service duplication, but not in service differentiation. The farther apart the rivals are, the more services are duplicated. With greater size asymmetry, a hospital utilizes its valuable resources for its own advantage to thwart competition from rivals by differentiating more high-tech services and reducing service duplication. It is noted that local market competition is not associated with service differentiation, but it is associated negatively with service duplication. It is possible that as competition becomes intense, hospitals avoid duplication of services for fear of a price war.

The findings indicate that high population density influences only differentiation of services, which is consistent with the hypothesis. Hospitals in a high-density community may need to add more high-tech services into their service lines. Hospitals in a community with high munificence offer more service duplication and less service differentiation. It is likely that hospitals take advantage of financial resources available by duplicating many high-tech services to the extent that there are not many services left for service differentiation. Other market factors also affect service duplication and differentiation. Hospitals respond to the demand of specialist physicians by adding the latest high-tech services [49]. Managed care tends to require that hospitals be a one-stop health service shopping for their members [50].

As more high-tech services are duplicated, the market share for each hospital on average becomes smaller. Losing market share not only raises average costs but also leads to lower profits unless hospitals are able to provide services in large quantities. Regarding service differentiation, the market share for each hospital on average does not become larger as hypothesized. As market share is shrinking, differentiation has the same impact on performance as duplication. It is likely that hospitals face difficulty in expanding market growth in differentiated services because these high-tech services might be too expensive for consumers to purchase.

The findings indicate that the market structure acts as a delicate check and balance on strategic duplication and differentiation. Hospitals respond to the market in a way that synchronizes between duplication and differentiation. When a hospital duplicates some services, it reduces differentiation of others, and vice versa. The market serves as a guide to signal hospitals which strategy should be implemented and which is demonstrated to be an excellent mechanism to gear the hospital to consumer needs.

Some types of hospitals tend to overdo duplication and differentiation, resulting in excess hospital capacity, particularly in markets with non-profit hospitals and hospitals joining multihospital systems that represent .776 and .806 of all hospitals, respectively (Table 3). Competing with rivals by duplicating services proves to be unwise in terms of performance, as this form of competition reduces market share and raises the possibility of a price war. Competing by differentiation does not do any better. As hospital markets have been flooded with more services than they need, adding more services either through duplication or differentiation proves to be bad strategies that raise costs and hurt the bottom line. Hospital management should analyze the supply and demand of the services they are providing and make adjustments accordingly for their service lines. It might be wise to cut back some services that are oversupplied and/or not profitable. Duplication and differentiation should be used only when demand for the services is greater than the supply.

## Limitations

One limitation of this study is related to market boundary. The use of county as a local market boundary might be imprecise for counting duplicated and differentiated services. It might also affect the measures of market competition. A second limitation is related to measuring managed care market penetration at the state level due to a lack of data available at the county level. Third, a cross-sectional design constrains the attribution of causality. Longitudinal studies and more refined market boundaries would enhance the findings.

## Conclusion

Caught in a dyadic competition with each of many rivals in a web of complex dynamic market, it is not easy for hospitals to find a proper strategy choosing between duplication and differentiation that will work simultaneously against many or all of their rivals. Overwhelmed and under pressure to compete, hospitals tend to provide more services, resulting in a market that is flooded with more services than it needs. To avoid this stressful situation, hospital management must identify a strategic fit between the internal organization and the external market before selecting strategies that are suitable with this fit. Analyzing the supply and demand of the services in the market and finding a balance between duplication and differentiation would help hospitals avoid a situation of excess capacity beyond the public needs that consequentially lead to rising costs and lower profits.

## Data Availability

The data used in this study was authorized by Margaret Weglarz under the license agreement with the American Hospital Association, but there are restrictions to the use of these data, which are not publicly available.

## Abbreviations

MEG: Magnetoencephalography
CBSAs: Core based statistical areas
AHA: American hospital association
CMS: Centers for medicare and medicaid services
SQL: Structured query language
ArcGIS: Geographic information system
HHI: Herfindahl-hirschman index
LISREL: Linear structural relations
GFI: Goodness-of-fit
AGFI: Adjusted goodness-of-fit
RMSR: Root mean square residual
